# Emotional Eating and Its Associations with the Prevalence of Depression and Anxiety Symptoms in University Students: A Cross-Sectional Study

**DOI:** 10.3390/medsci14030376

**Published:** 2026-07-06

**Authors:** Olga Alexatou, Sousana K. Papadopoulou, Exakousti-Petroula Angelakou, Athanasios Migdanis, Aikaterini Louka, Ioannis Migdanis, Maria Mentzelou, Theodosios Koimtsidis, Evmorfia Psara, Constantinos Giaginis

**Affiliations:** 1 Department of Food Science and Nutrition, School of the Environment, University of the Aegean, 81400 Lemnos, Greece; fnsd23003@fns.aegean.gr (O.A.); xeniaggelakou@hotmail.com (E.-P.A.); loukathy612@gmail.com (A.L.); maria.mentzelou@hotmail.com (M.M.); fnsd21013@fns.aegean.gr (E.P.); 2 Department of Nutritional Sciences and Dietetics, Faculty of Health Sciences, International Hellenic University, 57400 Thessaloniki, Greece; souzpapa@gmail.com (S.K.P.); koimtsidis1@gmail.com (T.K.); 3 Laboratory of Clinical Nutrition and Dietetics (CND-Lab), Department of Nutrition and Dietetics, University of Thessaly, 42132 Trikala, Greece; amigdanis@gmail.com; 4 Faculty of Medicine, University of Thessaly, 41110 Larissa, Greece; imigdanis@uth.gr; 5 Oncology Clinic and Hemodialysis Unit, “E. Patsidis” Health Group, Department of Clinical Nutrition, 41335 Larissa, Greece

**Keywords:** emotional eating, university students, psychological distress, depression, anxiety, mental health

## Abstract

**Background/Objectives:** Emotional eating (EE) is an emerging public health concern among university students, a population exposed to heightened academic demands, psychosocial stressors, and lifestyle changes that may promote maladaptive coping behaviors. EE has been linked to psychological distress, particularly depressive and anxiety symptoms, as well as sociodemographic, lifestyle, and anthropometric factors; however, findings remain heterogeneous and insufficiently integrated within comprehensive analytical frameworks. This study aimed to examine the association between EE and depressive and anxiety symptoms in university students, while assessing the independent contributions of sociodemographic, academic, lifestyle, and anthropometric determinants. **Methods:** A cross-sectional survey was performed among 1279 university students from 10 regions in Greece. Sociodemographic, academic, lifestyle, and anthropometric data were collected using validated instruments and standardized procedures. Depressive and anxiety symptoms were assessed by the use of the Beck Depression Inventory-II (BDI-II) and the six-item State-Trait Anxiety Inventory (STAI-6), respectively. EE was evaluated utilizing the EE subscale of the Three-Factor Eating Questionnaire–Revised 18 (TFEQ-R18). Multivariable ordinal logistic regression models were applied to examine independent associations. **Results:** In fully adjusted models, depressive and anxiety symptoms were the strongest correlates of higher EE levels, each associated with more than twofold increased odds. Female sex, Greek nationality, rural residence, enrollment in biomedical sciences, later academic years, and regular smoking were also positively associated with EE. Higher physical activity was inversely associated with EE levels. Overweight, obesity, and increased waist circumference and waist-to-hip ratio were consistently linked to higher EE, with several associations exceeding twofold increased odds. **Conclusions:** EE in university students is strongly associated with psychological distress and clusters with adverse lifestyle and anthropometric characteristics. These findings support the need for integrated interventions targeting mental health, lifestyle behaviors, and obesity-related risk factors. Longitudinal studies are warranted to clarify causal pathways and underlying mechanisms.

## 1. Introduction

The transition to university life represents a critical developmental period characterized by increased academic demands, lifestyle changes, and psychosocial stressors worldwide, including Greece [1,2]. This phase is often accompanied by greater independence in food choices, irregular meal patterns, and limited time for structured eating habits, all of which may negatively influence dietary behaviors [3]. Additionally, exposure to new social environments and financial constraints may further shape students’ food-related decisions and coping mechanisms [4]. These challenges may predispose students to maladaptive coping strategies, including unhealthy eating behaviors. Among these, emotional eating (EE)—defined as the tendency to consume food in response to negative emotions rather than physiological hunger—has gained increasing attention as a significant public health concern, particularly within young adult populations [5]. Notably, EE is often characterized by increased consumption of highly palatable, energy-dense foods rich in sugar and fat, which may provide temporary emotional relief but contribute to long-term adverse health outcomes [6]. Over time, the habitual reliance on food for emotional regulation may disrupt normal hunger and satiety cues, reinforcing unhealthy behavioral patterns [7]. Such hypotheses remain to be established by well-organized clinical trials.

Young adults such as university students constitute a vulnerable group with respect to mental health, with high prevalence rates of depressive and anxiety symptoms reported globally [8,9]. Recent meta-analyses have estimated pooled prevalences of depressive symptoms ranging from 27% to 34% and anxiety symptoms approaching 40%, highlighting the substantial psychological burden experienced by this population [8,9,10,11,12]. Factors such as academic pressure, uncertainty about future career prospects, and social adjustment difficulties may exacerbate psychological distress in this population [2,10]. Psychological distress during this life stage has been consistently linked to behavioral patterns that may adversely affect both physical and mental well-being [11]. EE has been proposed as one such coping mechanism, through which individuals attempt to regulate negative emotional states like melancholy, stress, or anxiety [5,12]. This behavior may be further reinforced by neurobiological pathways involving reward sensitivity and stress-related hormonal responses, such as cortisol dysregulation [13,14]. However, this behavior may contribute to a cycle of poor dietary choices, weight gain, and further psychological burden [14,15]. Importantly, this bidirectional relationship suggests that EE may both result from and contribute to worsening mental health outcomes [15,16]. However, epidemiological studies remain scarce so far, while there is not data at all concerning young populations such as university students in Greece.

Emerging evidence suggests a strong association between EE and mental health conditions, particularly depression and anxiety [12,14,15,16]. Individuals experiencing depressive symptoms may engage in EE as a form of self-soothing, often preferring energy-dense, palatable foods [17]. In some cases, EE may serve as a temporary distraction from negative cognitions, thereby reinforcing the behavior through short-term emotional relief [18]. Similarly, anxiety has been linked to dysregulated eating patterns, potentially mediated by heightened stress responses and altered appetite regulation [19]. Chronic activation of stress pathways may impair decision-making processes related to food choices, leading to impulsive or compulsive eating behaviors [13]. Despite growing interest in this area, findings remain inconsistent, especially in young populations, while the interplay between EE and psychological factors in university populations requires further investigation [16]. Differences in study design, measurement tools, and cultural contexts may partially explain these inconsistencies. With this regard, studies exploring the association between EE and mental health conditions, particularly depression and anxiety, remain scarce in young population from Greece.

In addition to psychological determinants, emotional eating has been associated with a range of sociodemographic, lifestyle, and anthropometric factors, including sex, physical activity, smoking behavior, and body weight status [20,21,22]. For instance, female students have frequently been reported to exhibit higher levels of EE, potentially due to gender differences in emotional processing and coping strategies [21]. Lifestyle behaviors such as physical inactivity and smoking may further compound the risk, reflecting broader patterns of health-related behaviors [22]. Anthropometric indicators, including body mass index and central adiposity measures, have also been linked to EE, suggesting a potential role in the development of overweight and obesity [23]. Understanding these associations is essential for identifying at-risk subgroups and developing targeted interventions. However, few studies have simultaneously examined the independent contribution of all these factors together alongside mental health variables in a comprehensive analytical framework.

Given the increasing prevalence of both EE and mental health disorders among university students, there is a clear need to better understand their interrelationship and associated determinants. Moreover, evidence regarding EE among university students in Greece remains scarce. Therefore, the present cross-sectional survey designed to explore the association between EE and depressive and anxiety symptoms among Greek university students, while also examining the contribution of sociodemographic, academic, lifestyle, and anthropometric characteristics. Based on the existing literature, we hypothesized that: (i) higher levels of depressive and anxiety symptoms would be associated with greater EE; (ii) EE would be more prevalent among students exhibiting adverse lifestyle and anthropometric characteristics, including lower physical activity levels, smoking, overweight, and obesity; and (iii) these associations would remain significant after adjustment for potential confounding factors in multivariable analyses. By identifying key correlates of EE, this study seeks to contribute to the development of targeted strategies aimed at improving both psychological well-being and health-related behaviors among university students.

## 2. Methods

### 2.1. Study’s Sample

The present cross-sectional survey firstly recruited 1571 university students from 10 distinct areas of Greece, encompassing both urban and rural settings (Athens, Thessaloniki, Larissa, Kalamata, Kavala, Korinthos, Alexandroupolis, Patra, and the North and South Aegean regions). Eligibility was restricted to individuals actively enrolled in Greek higher education institutions at the time of data collection. Participant recruitment took place over an extended period, from March 2021 to October 2024.

**Figure 1:**
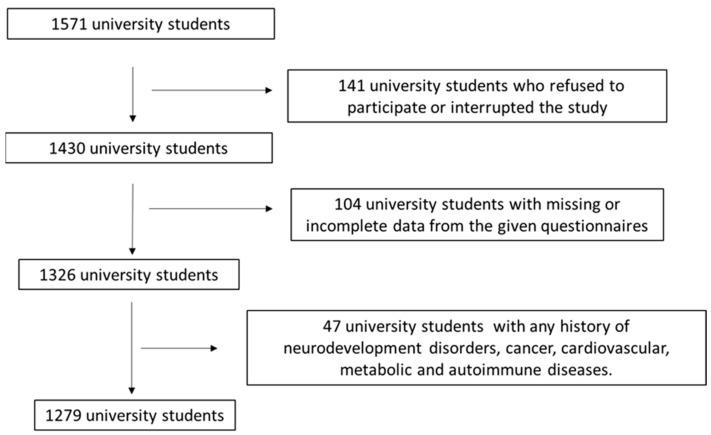
Flow chart diagram for study population enrollment.

Figure 1 illustrates the participant recruitment flowchart, including the application of predefined inclusion and exclusion criteria. Following eligibility screening, a total of 1279 active university students were retained for analysis, yielding a final response rate of 81.4%.

The survey protocol was approved by the Ethics Committee of the University of the Aegean (approval code: 21/11.10.2017; approval date: 11 October 2017) and was conducted in agreement with the ethical principles outlined in the World Medical Association Declaration of Helsinki (52nd WMA General Assembly, Edinburgh, Scotland, 2000). All participants provided written informed consent prior to enrollment, following a detailed explanation of the study objectives and procedures. Confidentiality and anonymity of participant data were strictly maintained throughout the study.

Individuals with self-reported histories of chronic conditions, including cardiometabolic diseases, psychiatric disorders, or malignancies, were excluded from participation to minimize potential confounding. Sample size estimation was performed a priori using PS: Power and Sample Size Calculation software, ensuring adequate statistical power for the planned analyses. Post hoc power analysis showed that the accomplished sample size provided a statistical power of 88.2% for the study’s primary analyses.

### 2.2. Assessment of Sociodemographic, Lifestyle and Anthropometric Factors

Suitable, validated questionnaires were administered to collect comprehensive information on the sociodemographic and lifestyle characteristics of assigned university students, including age, sex, nationality, place of residence, household economic status, living arrangements, parental marital status, smoking behavior, and employment status. In addition, given that all participants were actively enrolled in Greek higher education institutions, detailed academic information was obtained, including field of study, years of study, and academic performance. Data collection was conducted through structured face-to-face interviews between participants and qualified nutritionists and dietitians, in order to reduce recall biases and enhance data accuracy.

Household economic status was classified according to annual family income as follows: low (≤10,000 €), medium (>10,000 € and ≤20,000 €), and high (>20,000 €). Socioeconomic status was assessed using annual household income categories. The selected cut-offs were based on previous Greek epidemiological studies and are broadly consistent with income distributions reported by the Hellenic Statistical Authority (ELSTAT) and OECD classifications of household income groups [24]. Academic performance was categorized based on the official Greek grading system and the mean course grade at the time of assessment: low (5.0–6.49), moderate (6.5–8.49), and high (8.5–10.0), in accordance with the criteria of the Hellenic Ministry of Education.

Physical activity was assessed using the International Physical Activity Questionnaire (IPAQ), a widely validated instrument for population-based studies. Participants reported the frequency and duration of physical activity performed during a typical week. The IPAQ enables classification of physical activity levels into low, moderate, and high categories and has demonstrated acceptable reliability and validity across diverse populations, including both high- and middle-income countries. Total physical activity was expressed as a composite score incorporating walking, moderate, and vigorous activities over the previous seven days, calculated in MET-minutes per week (MET·min·wk^−1^) [25].

Anthropometric measurements, including body weight and height, were obtained under standardized conditions at the time of assessment. Weight was measured using a calibrated Seca digital scale (Seca, Hanover, MD, USA), with participants barefoot and wearing light clothing, and documented to the closest 100 g. Height was measured using a portable stadiometer (GIMA Stadiometer 27335, Gima S.p.A., Gessate, Italy), with participants barefoot, and documented to the closest 0.1 cm [26,27]. Body Mass Index (BMI) was calculated as weight in kilograms divided by height in meters squared (kg/m^2^) and categorized according to World Health Organization criteria as underweight (<18.5 kg/m^2^), normal weight (18.5–24.9 kg/m^2^), overweight (25.0–29.9 kg/m^2^), and obese (≥30.0 kg/m^2^) [26,27]. These standardized cut-offs are widely applied in epidemiological and clinical research to evaluate weight status and associated risks, including cardiovascular disease, type 2 diabetes mellitus, and mortality.

Waist circumference (WC) and waist-to-hip ratio (WHR), established indicators of central adiposity and cardiometabolic risk, were also measured using standardized protocols with a portable stadiometer (GIMA Stadiometer 27335), documented to the closest 0.1 cm. WC was categorized using internationally accepted cut-offs based on sex-specific thresholds (Men, Normal risk: <94 cm, Increased risk: 94–101.9 cm, High risk: ≥102 cm and Women, Normal risk: <80 cm, Increased risk: 80–87.9 cm, High risk: ≥88 cm) [28]. WHR was similarly classified into risk categories based on sex-specific thresholds (Men, Low risk–normal: WHR < 0.90, Moderate risk: WHR 0.90–0.99, High risk: WHR ≥ 1.00 and Women, Low risk–normal: WHR < 0.80, Moderate risk: WHR 0.80–0.84, High risk: WHR ≥ 0.85) [28]. The use of standardized anthropometric cut-offs enhances comparability across studies and supports consistent identification of individuals at elevated cardiometabolic risk, in line with World Health Organization recommendations [26,27].

For analytical purposes, continuous variables were transformed into categorical variables to facilitate interpretation, enable specific statistical modelling approaches, and identify clinically meaningful thresholds. From a clinical and epidemiological perspective, such categorization is often justified for improving interpretability, supporting decision-making processes, and enhancing the practical applicability of findings in public health and clinical contexts.

### 2.3. Assessment of Depression and Anxiety

Depressive symptoms were assessed using the Beck Depression Inventory–II (BDI-II), a widely utilized self-report instrument designed to evaluate the severity of depression [29]. The questionnaire comprises 21 items covering a broad range of affective, cognitive, motivational, and somatic manifestations of depression. These include emotional symptoms such as sadness and pessimism, cognitive aspects including self-criticism and feelings of worthlessness, as well as physical symptoms such as fatigue, changes in appetite, and diminished interest in sexual activity [29]. The BDI-II has been extensively validated across diverse populations and is recognized for its strong psychometric performance, including high reliability and construct validity, making it one of the most frequently employed instruments for the assessment of depressive symptom severity [29]. Each item is scored on a four-point scale ranging from 0 to 3, resulting in a total score between 0 and 63, with higher scores reflecting greater depressive symptomatology. The Greek adaptation of the BDI-II has demonstrated excellent psychometric characteristics, including high internal consistency (Cronbach’s α = 0.93) and satisfactory validity in both clinical and community-based populations [30]. In the present study, the instrument exhibited good internal reliability (Cronbach’s α = 0.856), supporting its suitability for evaluating depressive symptoms among university students.

Anxiety levels were measured using the six-item short form of the State-Trait Anxiety Inventory state scale (STAI-6). This abbreviated version was developed as a practical alternative to the original instrument and has been shown to provide reliable and valid estimates of state anxiety while substantially reducing respondent burden [31]. The STAI-6 evaluates core emotional states associated with anxiety, including feelings of tension, worry, distress, calmness, relaxation, and overall emotional well-being. Participants were asked to indicate the extent to which each statement reflected their current emotional condition. To facilitate comparability with the original STAI instrument, raw scores were converted to the standard STAI metric, yielding total scores ranging from 20 to 80 [31]. Based on established interpretative criteria, scores between 34 and 37 are considered representative of typical anxiety levels, whereas scores of 38 or higher indicate elevated anxiety symptomatology [32,33]. In the current study population, the STAI-6 demonstrated satisfactory internal consistency (Cronbach’s α = 0.823), confirming its appropriateness for assessing anxiety among university students.

### 2.4. Emotional Eating (EE) Evaluation

The Three-Factor Eating Questionnaire–Revised 18 (TFEQ-R18) was utilized to evaluate eating behavior patterns among participants [34,35]. The instrument comprises three theoretically derived subscales: (i) Cognitive Restraint, which reflects the conscious restriction of food intake to control body weight; (ii) Uncontrolled Eating, which describes the tendency to overeat with a perceived loss of control in response to internal or external food-related cues; and (iii) EE, which captures the propensity to eat in response to negative emotional states [34,35]. Higher scores on each subscale indicate stronger expression of the respective behavioral trait.

In the present study, the EE subscale was used, being further categorized into tertiles (low, moderate, and high emotional eating) to facilitate interpretability and enable meaningful group comparisons across analyses. The TFEQ-R18 EE subscale ranges from 3 (minimum score) to 12 (maximum score), which transforms to a 0–100 scale based on the following standard transformation formula:

$$Score=\frac{\left(S-L\right)}{R}\times 100$$

where S: individual raw subscale score, L: lowest possible raw score on that subscale, and R: possible raw score range (maximum − minimum).

The TFEQ-R18 has demonstrated strong psychometric performance across diverse populations. Its construct validity has been supported by confirmatory factor analysis, consistently confirming the three-factor structure. Internal consistency has been shown to be good to excellent, with Cronbach’s alpha coefficients typically ranging from approximately 0.70 to 0.90 across subscales, indicating acceptable to high reliability. Test–retest reliability has also been reported as satisfactory, supporting the stability of the instrument over time [34,35].

The Greek version of the TFEQ-R18 has been previously validated, demonstrating satisfactory internal consistency (Cronbach’s alpha values generally >0.70 across subscales), adequate construct validity, and good model fit indices in confirmatory factor analysis, thereby supporting its reliability and applicability in Greek-speaking populations [36]. These psychometric properties support the use of the TFEQ-R18 in the present study population.

### 2.5. Descriptive Statistics Presentation

The study included 1279 university students with a mean age of 20.5 ± 2.5 years (Table S1, Supplementary Material). Female participants comprised the majority of the sample (56.2%), while males accounted for 43.8%. Most students were of Greek nationality (81.9%), and slightly more than half resided in urban areas (52.7%). Regarding socioeconomic characteristics, 40.3% of participants reported low family economic status, 37.5% reported medium economic status, and 22.2% reported high economic status. More than half of the students were living with their families (55.7%), whereas 44.3% were living independently. Approximately one-third of participants reported having divorced parents (31.7%).

With respect to lifestyle characteristics, 32.2% of students were smokers and 35.6% were employed. Biomedical disciplines were the most common field of study, representing 54.8% of the sample, while 45.2% were enrolled in other academic disciplines. The distribution of students across years of study was relatively balanced, ranging from 24.6% in the first year to 25.8% in the fourth year. Nearly half of the participants reported good academic performance (46.8%), followed by very good (32.1%) and excellent performance (21.1%).

Physical activity levels were generally low, with nearly half of the students (49.7%) classified as having low physical activity, whereas 27.8% and 22.5% reported moderate and high physical activity levels, respectively. Based on BMI classification, 53.2% of participants had normal weight, 32.1% were overweight, and 14.7% were obese. WC assessment indicated that 61.4% of students were within the normal range, while 26.3% and 12.3% were classified as having increased and high cardiometabolic risk, respectively. Similarly, according to WHR, 65.6% were categorized as low risk, 26.1% as moderate risk, and 8.3% as high risk.

Regarding mental health indicators, depression was identified in 34.3% of participants, whereas anxiety was reported by 31.9%. The mean TFEQ-R18 EE score was 34.6 ± 21.5. Participants were evenly distributed across the EE tertiles, with 33.3% classified as having low EE, 33.4% moderate EE, and 33.3% high EE levels.

### 2.6. Statistical Analysis

Continuous variables were initially assessed for normality utilizing the Kolmogorov–Smirnov test. Variables following a normal distribution were analyzed utilizing the Student’s *t*-test, and are depicted as mean ± standard deviation (SD). Categorical variables are expressed as absolute frequencies and percentages, and group comparisons were performed by the chi-square test. Effect sizes for chi-square associations were estimated using Cramer’s V, with values of nearly 0.10, 0.30, and 0.50 considered indicative of small, moderate, and large effect sizes, respectively.

The Emotional Eating subscale of the Three-Factor Eating Questionnaire–Revised 18 (TFEQ-R18) was calculated according to standard scoring procedures. Given its non-normal distribution and to facilitate interpretation in relation to behavioral risk patterns, EE scores were categorized into tertiles (low, moderate, and high emotional eating). This categorization approach was applied to enable comparison across groups and to identify potential dose–response associations with outcome variables.

A multivariable ordinal logistic regression analysis was conducted to investigate the independent associations between university students’ EE tertiles and selected sociodemographic, and anthropometric factors. Consistent with the predefined analytical strategy, only variables exhibiting statistically significant associations with EE in the univariate analyses were entered into the multivariable ordinal logistic regression model to control for potential confounding effects. The results are presented as adjusted odds ratios (ORs) with corresponding 95% confidence intervals (CIs). For the multivariable ordinal logistic regression analysis, adjusted ORs and their corresponding 95% CIs were reported as measures of effect size.

Prior to model fitting, multicollinearity amongst independent variables was evaluated by variance inflation factors (VIFs), with values > 5 indicating potential collinearity concerns. No evidence of problematic multicollinearity was observed. Missing data was handled utilizing complete-case analysis, as the proportion of missing values was low (<5%) and considered to be missing at random.

The proportional odds assumption underlying the ordinal logistic regression model was assessed utilizing the Test of Parallel Lines, while overall model fit and statistical significance were assessed using the Likelihood Ratio Test.

Regarding sample size and statistical power, the total sample of 1279 participants was considered adequate for the planned multivariable analysis. Based on the rule of at least 10 events per parameter, and given the distribution of participants across EE tertiles, the study met the recommended criteria for stable model estimation. Furthermore, a post hoc power analysis was conducted assuming a two-sided α = 0.05, a proportion of exposure of approximately 30–50% (typical for categorical predictors such as gender or smoking), and an odds ratio of 1.30 (small-to-moderate effect size). Under these assumptions, the available sample size (n = 1279) provided statistical power exceeding 90% to detect significant associations in the ordinal logistic regression model. Even for smaller effect sizes (OR ≈ 1.20), the study retained acceptable power (approximately 80%), supporting the robustness of the findings.

All statistical tests were two-sided, and a *p*-value < 0.05 was considered indicative of statistical significance. Statistical analyses were performed using Statistica software, version 10.0 (Informer Technologies Inc., Hamburg, Germany).

## 3. Results

### 3.1. Association of EE with Sociodemographic, Anthropometric, Lifestyle and Behavioral Factors

Cross-tabulation analyses revealed several statistically significant associations between participant characteristics and levels of EE. Female participants exhibited significantly higher EE levels compared to males (Table 1, *p* < 0.0001). Likewise, individuals of Greek nationality demonstrated significantly elevated EE levels relative to participants of other nationalities (Table 1, *p* = 0.0001). Students reporting a rural permanent residence had significantly higher EE levels compared to those residing in urban areas (Table 1, *p* < 0.0001). Additionally, regular smokers showed a significantly higher incidence of elevated EE levels than non-smokers (Table 1, *p* < 0.0001).

With respect to academic variables, enrollment in biomedical science disciplines was significantly associated with higher EE levels compared to other fields of study (Table 1, *p* < 0.0001). Lower academic performance was also significantly correlated with increased EE levels (Table 1, *p* < 0.0001). Furthermore, students in the earlier stages of their academic programs exhibited significantly higher EE levels than those in later years (Table 1, *p* < 0.0001).

Lifestyle and anthropometric factors were similarly associated with EE. Lower levels of physical activity were significantly linked to higher EE levels (Table 1, *p* < 0.0001). Overweight and obese participants demonstrated significantly greater EE levels than those with normal body weight (Table 1, *p* < 0.0001). Measures of central adiposity were also positively associated with EE, as indicated by significantly higher EE levels among students with elevated WC and WHR (Table 1, *p* < 0.0001 for both). Moreover, the presence of depressive symptoms was significantly associated with higher EE levels (Table 1, *p* < 0.0001), and a similar pattern was observed for anxiety symptoms, with affected students exhibiting significantly higher EE levels than those without such symptoms (Table 1, *p* < 0.0001).

In contrast, no statistically significant associations were identified between EE levels and age, family economic status, living arrangements, parental status, or employment status (Table 1, *p* > 0.05).

Effect size analysis using Cramer’s V indicated that most statistically significant associations between EE and sociodemographic or lifestyle characteristics were of small magnitude (V = 0.111–0.234). The strongest associations were observed for BMI (V = 0.376), depression (V = 0.352), waist circumference (V = 0.335), and anxiety (V = 0.320), indicating moderate effect sizes. In contrast, family economic status (V = 0.049) and employment status (V = 0.023) demonstrated negligible associations with EE.

### 3.2. Multivariate Ordinal Logistic Regression Analysis for Emotional Eating (EE) of the Study Population

Multivariable ordinal logistic regression analysis indicated that EE levels were independently associated with a range of sociodemographic, academic, lifestyle, anthropometric, and psychological factors (Table 2, *p* < 0.05 for all). Specifically, female sex was significantly associated with higher EE levels, with female students exhibiting greater odds of belonging to higher EE categories compared to males (OR = 1.51; *p* = 0.0071). Greek nationality was likewise positively associated with EE, as Greek students had a 23% higher likelihood of elevated EE levels relative to non-Greek participants (OR = 1.23; *p* = 0.0056).

Residence in rural areas was also significantly associated with increased EE, with these students demonstrating a 49% higher likelihood of belonging to higher EE categories compared to those residing in urban settings (OR = 1.49; *p* = 0.0012). With regard to academic characteristics, enrollment in biomedical sciences was significantly associated with elevated EE levels (OR = 1.27; *p* = 0.0031). In addition, students in the earlier years of study (first and second year) exhibited higher odds of elevated EE compared with those in the later years of study (third and fourth year) (OR = 1.19; *p* = 0.0052).

In terms of lifestyle factors, regular smoking was significantly associated with higher EE levels, corresponding to a 64% increase in the odds of belonging to higher EE categories (OR = 1.64; *p* = 0.0008). Conversely, higher levels of physical activity were inversely associated with EE, as students with high physical activity demonstrated a 43% reduction in the odds of higher EE levels compared to those with low physical activity (OR = 0.57; *p* = 0.0002).

Anthropometric indicators showed strong and consistent associations with EE. Obesity, as defined by body mass index (BMI), was significantly associated with EE, with obese students exhibiting more than a twofold increase in the odds of higher EE levels compared to normal-weight individuals (OR > 2.00; *p* = 0.0003). Similarly, overweight status was associated with nearly twofold increased odds of elevated EE levels (OR = 1.98; *p* = 0.0001).

Comparable patterns were observed for measures of central adiposity. Students classified as high risk based on WC had approximately twice the odds of higher EE levels compared to those at low risk (OR = 1.97; *p* = 0.0006). Likewise, individuals categorized as high risk according to WHR exhibited more than a twofold increase in the odds of higher EE levels relative to those with normal WHR values (OR = 2.11; *p* = 0.0001).

Psychological factors were also strongly associated with EE. The presence of depressive symptoms was associated with more than double the odds of higher EE levels (OR = 2.45; *p* = 0.0002), while anxiety symptoms were similarly associated with increased EE, with affected students demonstrating more than twice the odds of higher EE levels compared to those without anxiety (OR = 2.31; *p* = 0.0003).

In contrast, no statistically significant differences were detected between intermediate and reference categories of central adiposity (i.e., increased-risk WC vs. normal WC, and moderate-risk WHR vs. normal WHR) (*p* > 0.05). Additionally, academic performance was not significantly associated with EE levels in the multivariable model (Table 2, *p* > 0.05). The proportional odds assumption was satisfied (Test of Parallel Lines, *p* = 0.45), and the overall model demonstrated good fit and statistical significance (Likelihood Ratio Test, *p* < 0.001).

## 4. Discussion

The present cross-sectional study systematically examined the associations between EE levels and an extensive array of sociodemographic, academic, lifestyle, anthropometric, and psychological variables within a university student population, with particular emphasis on depressive and anxiety symptomatology. Overall, the findings suggest that EE is situated within a broader nexus of psychological distress and health-related behaviors, with depressive and anxiety symptoms emerging as the most salient and consistent correlates of elevated EE across both univariate and multivariable analytical models.

A central finding of this investigation is the robust and independent association between emotional eating and psychological distress, specifically depression and anxiety symptoms. Students reporting depressive symptomatology demonstrated more than a twofold increase in the likelihood of elevated EE, while those exhibiting anxiety symptoms showed a comparably heightened risk. These results are congruent with an extensive body of literature indicating that EE is closely linked to internalizing forms of psychopathology among young adults [2,14]. Existing systematic reviews and meta-analyses have consistently documented positive associations between depression, anxiety, stress, and EE, particularly within university populations, which are widely recognized as being at increased risk for mental health challenges [2,11,15]. This association is commonly interpreted through the lens of affect regulation models, which posit that individuals may engage in eating behaviors—especially the consumption of highly palatable, energy-dense foods—as maladaptive strategies to modulate negative emotional states [16,18,37]. Although such behaviors may confer short-term emotional relief, they are likely to perpetuate a maladaptive cycle in which eating is repeatedly employed as a coping mechanism, thereby sustaining both psychological distress and dysregulated eating patterns over time [7,18,37].

The strength and independence of these associations further lend support to neurobehavioral conceptualizations of EE. Depression and anxiety have been linked to dysregulation in reward-processing neural circuits and alterations in hypothalamic–pituitary–adrenal (HPA) axis functioning, which may enhance responsiveness to food-related reward stimuli while simultaneously compromising inhibitory control over eating behaviors [7,38,39]. These mechanisms provide a plausible biological basis for the observed propensity among psychologically distressed individuals to overconsume foods high in sugar and fat. Moreover, a growing body of evidence supports a bidirectional relationship between EE and psychological distress, whereby EE may not only arise as a consequence of depressive and anxiety symptoms but may also contribute to their maintenance and exacerbation through pathways involving weight gain, body image dissatisfaction, and further emotional dysregulation [38,40,41].

In addition to psychological determinants, several sociodemographic factors were independently associated with EE. Female students exhibited significantly higher levels of EE than their male counterparts, a finding consistent with prior research indicating greater emotional reactivity, higher prevalence of internalizing symptoms, and gender-specific coping strategies among women [42,43]. Sociocultural pressures pertaining to body image and eating behaviors may further account for these observed sex differences. Furthermore, Greek nationality and rural residence were associated with increased levels of EE. Although empirical evidence regarding cultural and geographical variability in EE among university students remains relatively limited, existing studies suggest that environmental stressors, dietary environments, and sociocultural norms may play a role in shaping such behaviors [44,45,46]. Rural residence, in particular, may be linked to differences in food accessibility, social support networks, and lifestyle patterns that indirectly influence eating behavior.

Lifestyle-related behaviors were also consistently associated with EE across analytical models. Regular smoking was positively associated with higher EE levels, whereas engagement in physical activity demonstrated a protective effect. These findings align with the broader conceptualization of EE as part of a clustering of health-risk behaviors [11,37,47]. Previous studies have similarly indicated that EE frequently co-occurs with sedentary lifestyles and substance use, suggesting shared underlying mechanisms such as heightened stress reactivity and deficits in self-regulatory capacity. The inverse association between physical activity and EE is particularly noteworthy, given the well-established role of physical activity in enhancing mood regulation and attenuating stress responses, thereby potentially mitigating emotionally driven eating [11,37,47].

Anthropometric outcomes further underscore the clinical significance of EE. Overweight and obese individuals, as well as those presenting with elevated WC and WHR, were significantly more likely to report higher levels of EE [48,49]. These findings are consistent with longitudinal and meta-analytic evidence demonstrating a positive association between EE and increased BMI as well as central adiposity [47,48,49]. Notably, it should be noted that causal inferences cannot be derived from the present cross-sectional design; however, these associations lend support to the hypothesis that EE may contribute to excess caloric intake and subsequent weight gain [7,50,51]. Conversely, increased adiposity may exacerbate psychological distress and reinforce maladaptive eating behaviors, thereby supporting a bidirectional relationship between EE and body weight [52].

With respect to academic characteristics, the findings were somewhat heterogeneous. Students enrolled in biomedical disciplines and those in the earlier years of study exhibited significantly higher levels of EE compared with students enrolled in other academic disciplines and those in later years of study, respectively, whereas academic performance did not retain statistical significance in adjusted models. These results may reflect the cumulative burden of academic stress and workload rather than objective academic achievement per se [53,54]. Consistent with this interpretation, prior research has indicated that perceived stress is a more salient predictor of disordered eating behaviors than academic performance indicators [8,55].

In contrast, no statistically significant associations were observed between EE and age, family economic status, living arrangements, parental status, or employment status. These null findings suggest that, within a relatively homogeneous university cohort, proximal psychological and behavioral factors may exert a more pronounced influence on emotional eating than broader structural or socioeconomic determinants.

The study possesses several notable methodological strengths. It employed a relatively large sample and adopted a comprehensive analytical framework encompassing multiple domains, thereby facilitating the identification of independent correlates of EE while minimizing potential confounding. The use of multivariable ordinal logistic regression enabled robust estimation of associations and the examination of graded relationships across levels of EE, enhancing the internal validity of the findings. Additionally, the incorporation of validated psychometric instruments for assessing depressive and anxiety symptoms strengthens the reliability of the observed associations. The concurrent evaluation of general and central adiposity indicators—including BMI, WC, and WHR ratio—further provides a more nuanced assessment of body composition than reliance on a single anthropometric measure.

Nevertheless, several limitations should be acknowledged. The cross-sectional nature of the study precludes causal inference and limits the ability to determine temporal sequencing between emotional eating and psychological distress. As such, it remains unclear whether depressive and anxiety symptoms act as antecedents or consequences of EE, or whether the relationship is inherently bidirectional. The reliance on self-reported data introduces the potential for information bias, including recall and social desirability biases, particularly with respect to dietary behaviors, anthropometric measures, and mental health indicators. Although validated instruments were utilized, self-report methodologies are inherently susceptible to measurement error. Furthermore, the exclusive focus on a university student population may constrain the generalizability of the findings to other demographic groups. Moreover, despite adjustment for a wide range of covariates, the possibility of residual confounding cannot be entirely excluded, particularly in relation to unmeasured factors such as dietary quality, sleep patterns, personality characteristics, and genetic influences. Finally, an additional limitation is that scale reliability was assessed using Cronbach’s alpha only, whereas contemporary psychometric recommendations often advocate reporting complementary reliability indices such as McDonald’s omega. Although the large sample size (n = 1279) and satisfactory Cronbach’s alpha coefficients support the robustness of the findings, future studies should consider the use of multiple reliability indices to provide a more comprehensive assessment of internal consistency.

## 5. Conclusions

The present study underscores EE as a multidimensional construct that is closely and consistently associated with psychological distress and a range of health-related behaviors among university students. Depressive and anxiety symptomatology emerged as the most stable and robust correlates, highlighting the central role of affective dysregulation and emotional control processes in the modulation of eating behavior within this population. These findings are in accordance with established theoretical frameworks that conceptualize EE as a maladaptive coping strategy, as well as neurobehavioral models that implicate alterations in reward processing and deficits in self-regulatory control in the context of psychological distress.

In addition to psychological determinants, EE was independently associated with several sociodemographic and lifestyle factors, including female sex, tobacco use, lower levels of physical activity, and indicators of increased adiposity. The observed clustering of EE with other maladaptive health behaviors and unfavorable anthropometric profiles further underscores its clinical significance and public health relevance. Conversely, the absence of associations with several socioeconomic indicators suggests that, within relatively homogeneous university populations, proximal psychological and behavioral factors may exert a more pronounced influence on EE than distal structural determinants.

Collectively, these findings support the need for a comprehensive and integrative approach to the identification and management of EE in university settings. Interventions should prioritize the early detection and treatment of depressive and anxiety symptoms, while simultaneously fostering adaptive emotion regulation strategies, increasing physical activity levels, and promoting broader healthy lifestyle behaviors. Embedding such preventive and therapeutic strategies within university health and counseling services may be particularly effective in reducing the potential long-term consequences of EE, including excess weight gain and the persistence of psychological distress.

Future research should employ longitudinal designs to better elucidate the temporal directionality and potential bidirectional relationships underlying these associations. Furthermore, the inclusion of objective behavioral, physiological, and biological measures, alongside psychosocial assessments, would enhance mechanistic understanding of EE. Despite inherent limitations, the present study contributes to the expanding literature by reinforcing the conceptualization of EE as a critical interface between mental health and lifestyle-related behaviors in young adult populations.

## Figures and Tables

**Table 1:** Associations of Emotional Eating (EE) with sociodemographic, anthropometric, lifestyle and psychological factors of the assigned university students.

Variables (n = 1279)	Emotional Eating (EE)				
	Low 426 (33.3%)	Medium 427 (33.4%)	High 426 (33.3%)	*p*-Value	Cramer’s V
**Age (mean ± SD; years)**	20.4 ± 2.3	20.5 ± 2.5	20.2 ± 2.4	*p* = 0.4093	
**Gender (n, %)**				*p* ˂ 0.0001	0.141
Male	228 (53.5%)	160 (37.5%)	172 (40.4%)		
Female	198 (46.5%)	267 (62.5%)	254 (59.6%)		
**Nationality (n, %)**				*p* = 0.0001	0.119
Greek	323 (75.8%)	371 (86.9%)	353 (82.9%)		
Other	103 (24.2%)	56 (13.1%)	73 (17.1%)		
**Type of residence (n, %)**				*p* ˂ 0.0001	0.139
Urban	263 (61.7%)	220 (51.5%)	191 (44.8%)		
Rural	163 (38.3%)	207 (48.5%)	235 (55.2%)		
**Family economic status (n, %)**				*p* = 0.1805	0.049
Low	174 (40.9%)	163 (38.2%)	179 (42.0%)		
Medium	167 (39.2%)	152 (35.6%)	160 (37.6%)		
High	85 (19.9%)	112 (26.2%)	87 (20.4%)		
**Living status (n, %)**				*p* = 0.1929	0.126
Living with family	204 (47.9%)	270 (63.2%)	238 (55.9%)		
Living alone	222 (52.1%)	157 (36.8%)	188 (44.1%)		
**Parents marital status (n, %)**				*p* = 0.0801	0.115
No divorced	272 (63.9%)	324 (75.9%)	278 (65.3%)		
Divorced	154 (36.1%)	103 (24.1%)	148 (34.7%)		
**Smoking status (n, %)**				*p* ˂ 0.0001	0.181
No smokers	328 (77.0%)	298 (69.8%)	241 (56.6%)		
Regular smokers	98 (23.0%)	129 (30.2%)	185 (43.4%)		
**Type of Studies (n, %)**				*p* ˂ 0.0001	0.181
Biomedical studies	186 (43.7%)	281 (65.8%)	234 (54.9%)		
Other studies	239 (56.3%)	146 (34.2%)	192 (45.1%)		
**Years of Studies (n, %)**				*p* ˂ 0.0001	0.234
One year	92 (21.6%)	45 (10.5%)	178 (41.8%)		
Two years	108 (25.3%)	102 (23.9%)	106 (24.9%)		
Three years	95 (22.3%)	140 (32.8%)	83 (19.5%)		
Four years	131 (30.8%)	140 (32.8%)	59 (13.8%)		
**Academic performance (n, %)**				*p* ˂ 0.0001	0.131
Good	203 (47.8%)	153 (35.8%)	243 (57.0%)		
Very good	122 (28.7%)	176 (41.1%)	112 (26.3%)		
Excellent	100 (23.5%)	99 (23.1%)	71 (16.7%)		
**Employment status (n, %)**				*p* = 0.7192	0.023
No employee	281 (66.0%)	272 (63.7%)	271 (63.6%)		
Employee	145 (34.0%)	155 (36.3%)	155 (36.4%)		
**Physical activity (n, %)**				*p* ˂ 0.0001	0.150
Low	152 (35.8%)	226 (52.8%)	258 (60.6%)		
Medium	157 (36.9%)	102 (23.8%)	96 (22.5%)		
High	116 (27.3%)	100 (23.4%)	72 (16.9%)		
**BMI (n, %)**				*p* ˂ 0.0001	0.376
Normal weight	313 (73.6%)	329 (76.9%)	85 (19.9%)		
Overweight	93 (21.9%)	81 (18.9%)	249 (58.5%)		
Obese	19 (4.5%)	18 (4.2%)	92 (21.6%)		
**WC (n, %)**				*p* ˂ 0.0001	0.335
Normal	326 (76.7%)	337 (78.7%)	123 (28.9%)		
Increased risk	71 (16.7%)	60 (14.0%)	205 (48.1%)		
High risk	28 (6.6%)	31 (7.2%)	98 (23.0%)		
**WHR (n, %)**				*p* ˂ 0.0001	0.111
Low risk	295 (69.4%)	297 (69.4%)	247 (58.0%)		
Moderate risk	91 (21.4%)	116 (27.1%)	127 (29.8%)		
High risk	39 (9.2%)	15 (3.5%)	52 (12.2%)		
**Depression (n, %)**				*p* ˂ 0.0001	0.352
No	352 (82.8%)	305 (71.3%)	183 (43.0%)		
Yes	73 (17.2%)	123 (28.7%)	243 (57.0%)		
**Anxiety (n, %)**				*p* ˂ 0.0001	0.320
No	362 (85.2%)	301 (70.3%)	208 (48.8%)		
Yes	63 (14.8%)	127 (29.7%)	218 (51.2%)		

**Table 2:** Multivariate ordinal logistic regression model of factors associated with emotional eating (EE).

Variables	Comparison	Reference Category *	Adjusted OR **	95% CI ***	*p*-Value
Gender	Female	Male	1.51	1.31–1.72	0.0071
Nationality	Greek	Other	1.23	0.98–1.37	0.0056
Type of residence	Rural	Urban	1.49	1.34–1.71	0.0012
Smoking status	Regular smokers	No smokers	1.64	1.47–1.92	0.0008
Type of Studies	Biomedical studies	Other studies	1.27	1.09–1.1.45	0.0031
Years of Studies	1st + 2nd	3rd + 4th	1.19	0.96–1.35	0.0052
Academic performance	Good	Very good	1.09	0.68–1.57	0.2981
	Good	Excellent	1.15	0.80–1.47	0.1802
Physical activity	Medium	Low	0.81	0.61–1.15	0.0102
	High	Low	0.57	0.41–0.75	0.0002
BMI	Overweight	Normal	1.98	1.80–2.11	0.0003
	Obese	Normal	2.21	2.01–2.37	0.0001
WC	Increased risk	Normal risk	1.34	0.92–1.99	0.2763
	High risk	Normal risk	1.97	1.81–2.14	0.0006
WHR ratio	Moderate risk	Normal risk	1.30	0.72–1.95	0.3987
	High risk	Normal risk	2.11	1.92–2.25	0.0001
Depression	Yes	No	2.45	2.29–2.62	0.0002
Anxiety	Yes	No	2.31	2.14–2.51	0.0003

* Reference categories used in the ordinal logistic regression analysis were: male sex, non-Greek nationality, urban residence, non-smoking status, other studies, first/second year of studies, very good or excellent academic performance (as indicated), low physical activity, normal weight, normal waist circumference, low-risk waist-to-hip ratio, absence of depression, and absence of anxiety. ** ORs: Odds Ratios, *** CIs: Confidence Intervals.

## Data Availability

The original contributions presented in this study are included in the article/Supplementary Material. Further inquiries can be directed to the corresponding author.

## Supplementary Materials

The following supporting information can be downloaded at: https://www.mdpi.com/article/10.3390/medsci14030376/s1, Table S1: Descriptive statistics of the enrolled university students.
