# Supplementary material for: Emotional Eating and Its Associations with the Prevalence of Depression and Anxiety Symptoms in University Students: A Cross-Sectional Study
Source: Med Sci (Basel). 2026 Jul 6;14(3):376. doi: 10.3390/medsci14030376 (PMC13414101; doi:10.3390/medsci14030376)
Supplement: Supplementary file 1 [file medsci-14-00376-s001.zip › medsci-4422247-supplementary.pdf]

Table S1. Descriptive statistics of the enrolled university students.

**Variables (n=1279)**	**Descriptive statistics**
**Age (mean±SD; years)**	20.5±2.5
**Gender (n, %)**	
Male	560 (43.8%)
Female	719 (56.2%)
**Nationality (n, %)**	
Greek	1047 (81.9%)
Other	232 (18.1%)
**Type of residence (n, %)**	
Urban	674 (52.7%)
Rural	605 (47.3%)
**Family economic status (n, %)**	
Low	516 (40.3%)
Medium	479 (37.5%)
High	284 (22.2%)
**Living status (n, %)**	
Living with family	712 (55.7%)
Living alone	567 (44.3%)
**Parents marital status (n, %)**	
No divorced	874 (68.3%)
Divorced	405 (31.7%)
**Smoking status (n, %)**	
No smokers	867 (67.8%)

Smokers	412 (32.2%)
**Type of Studies (n, %)**	
Biomedical studies	701 (54.8%)
Other studies	578 (45.2%)
**Years of Studies (n, %)**	
One year	315 (24.6%)
Two years	316 (24.7%)
Three years	318 (24.9%)
Four years	330 (25.8%)
**Academic performance (n, %)**	
Good	599 (46.8%)
Very good	410 (32.1%)
Excellent	270 (21.1%)
**Employment status (n, %)**	
Employee	455 (35.6%)
No employee	824 (64.4%)
**Physical activity (n, %)**	
Low	636 (49.7%)
Medium	355 (27.8%)
High	288 (22.5%)
**BMI (n, %)**	
Normal weight	680 (53.2%)
Overweight	411 (32.1%)
Obese	188 (14.7%)

**WC (n, %)**	
Normal	786 (61.4%)
Increased risk	336 (26.3%)
High risk	157 (11.3%)
**WHR (n, %)**	
Low risk	839 (65.6%)
Moderate risk	334 (26.1%)
High risk	106 (8.3%)
**Depression (n, %)**	
No	840 (65.7%)
Yes	439 (34.3%)
**Anxiety (n, %)**	
No	871 (68.1%)
Yes	408 (31.9%)
**TEFQ-R18 (mean ± SD)**	34.6 ± 21.5.
**TEFQ-R18 (Tertiles)**	
Low EE (Q1, n%)	426 (33.3%)
Moderate EE (Q2, n%)	427 (33.4%)
High EE (Q3, n%)	426 (33.3%)
